# Economic Costs of Providing District- and Regional-Level Surgeries in Tanzania

**DOI:** 10.34172/ijhpm.2021.09

**Published:** 2021-02-23

**Authors:** Martilord Ifeanyichi, Henk Broekhuizen, Adinan Juma, Kondo Chilonga, Edward Kataika, Jakub Gajewski, Ruairi Brugha, Leon Bijlmakers

**Affiliations:** ^1^Department for Health Evidence, Radboud Institute for Health Sciences, Radboud University Medical Centre, Nijmegen, The Netherlands.; ^2^East, Central and Southern Africa Health Community, Arusha, Tanzania.; ^3^Department of Surgery, Kilimanjaro Christian Medical Centre, Moshi, Tanzania.; ^4^Institute of Global Surgery, Royal College of Surgeons in Ireland, Dublin, Ireland.; ^5^Department of Epidemiology and Public Health Medicine, Royal College of Surgeons in Ireland, Dublin, Ireland.

**Keywords:** Global Surgery, Tanzania, District Hospital, Surgical Costs, Economies of Scale

## Abstract

**Background:** Access to surgical care is poor in Tanzania. The country is at the implementation stage of its first National Surgical, Obstetric, and Anesthesia Plan (NSOAP; 2018-2025) aiming to scale up surgery. This study aimed to calculate the costs of providing surgical care at the district and regional hospitals.

**Methods:** Two district hospitals (DHs) and the regional referral hospital (RH) in Arusha region were selected. All the staff, buildings, equipment, and medical and non-medical supplies deployed in running the hospitals over a 12 month period were identified and quantified from interviews and hospital records. Using a combination of step-down costing (SDC) and activity-based costing (ABC), all costs attributed to surgeries were established and then distributed over the individual types of surgeries. These costs were delineated into pre-operative, intra-operative, and post-operative components.

**Results:** The total annual costs of running the clinical cost centres ranged from $567k at Oltrumet DH to $3453k at Mt Meru RH. The total costs of surgeries ranged from $79k to $813k; amounting to 12%-22% of the total costs of running the hospitals. At least 70% of the costs were salaries. Unit costs and relative shares of capital costs were generally higher at the DHs. Two-thirds of all the procedures incurred at least 60% of their costs in the theatre. Open reduction and internal fixation (ORIF) performed at the regional hospital was cheaper ($618) than surgical debridement (plus conservative treatment) due to prolonged post-operative inpatient care associated with the latter ($1177), but was performed infrequently due mostly to unavailability of implants.

**Conclusion:** Lower unit costs and shares of capital costs at the RH reflect an advantage of economies of scale and scope at the RH, and a possible underutilization of capacity at the DHs. Greater efficiencies make a case for concentration and scale-up of surgical services at the RHs, but there is a stronger case for scaling up district-level surgeries, not only for equitable access to services, but also to drive down unit costs there, and free up RH resources for more complex cases such as ORIF.

## Background

**Key Messages**

** Implications for policy makers**

- Greater efficiencies and economies of scale and scope make the case for greater concentration and scaling up of the volume of surgical services at the referral hospital (RH). However, there is a stronger case for scaling up basic surgery at district hospitals (DHs), to increase equitable access to surgery and produce lower unit costs at the DH, also freeing up resources at the RH for more complex cases.
- While implants are prohibitively expensive for regional hospitals which have the technical capacity to perform open reduction and internal fixation (ORIF) of fractures, it does make economic sense to purchase them as the alternative management options incur high post-operative costs.
- Further interventions to scale up surgery at the DHs should prioritise less tangible rate limiting factors, such as staff skills, anaesthesia capacity, and supplies, over investments in physical infrastructure.
- To derive full benefits from the manpower available at DHs, disincentives for self-referrals to RHs (eg, introduction and enforcement of penalty fees), and a mechanism of continuing surgical training and supervision at the DHs (such as trialled in SURG-Africa) are needed.

** Implications for the public**

With the launch of a National Surgical, Obstetric, and Anesthesia Plan (NSOAP) in 2018, Tanzania embarked on an ambitious journey to make safe and timely surgery available to everybody by 2025. The findings in this paper show that surgery carried out at a referral hospital (RH) may be more efficient but that investments are needed to scale up surgery at district hospitals (DHs), to promote equitable access to surgery and ensure that resources at RHs are freed up to manage more complex cases.

Access to surgery remains poor in low- and middle-income countries (LMICs), where as much as 95% of the people do not have access to safe and timely surgical care.^1^ Only about 6% of all the surgeries performed globally are performed in LMICs,^2^ despite the fact that these populations bear the greatest needs for surgical care: 38 disability-adjusted life years (DALYs) per 1 million people are lost to conditions amenable to surgery annually in sub-Saharan Africa alone.^3^ These translate to a projected productivity loss of about US$12 trillion between 2015 and 2030.^2^ An estimated 7.2 million DALYs could be averted annually in LMICs by investing in essential surgical, obstetric and anaesthesia (SOA) care.^4^ There is a need for empirical evidence to guide such investments.

 Much is known about the required system-level financial investments for the scale-up of surgery in LMICs. Verguet et al estimated that achieving the target proposed by the Lancet Commission on Global Surgery (LCGS) of 5000 major surgical procedures per 100 000 persons per year would require between US$300 and US$420 billion.^5^ Noting that Verguet and colleagues’ study omitted the cost requirements for relevant human resource development, Jumbam et al reported the costs of implementation of National Surgical, Obstetric, and Anesthesia Plans (NSOAPs) in 4 African countries – Rwanda, Zambia, Tanzania and Nigeria – to be US$5.6, US$18.1, US$10.57, and US$85.6 per capita, respectively.^6^ Differences in NSOAP priorities, local costs of interventions and costing methodologies account for the wide variations in costs,^6^ making cross-country comparisons difficult.

 The resource requirements and costs of individual types of surgeries in LMICs are less well-explored however. Surgeries are technically complex, and some of their costs are shared with other health services. Much of the associated costs may therefore be masked.^7^ This makes costing studies particularly relevant. In SSA, only few such studies have been reported: on caesarean sections (CSs)^8^ and laparotomies^9^ in Rwanda; open heart surgeries in Nigeria^10^; neurosurgical management of infant hydrocephalus in Africa^11^; burn care in sub-Saharan Africa^12^; and common surgeries in Uganda.^13^ In Tanzania, to the best of our knowledge, only one study has investigated the cost of a major type of surgery: Open reduction and internal fixation (ORIF).^14^ That study took a tertiary hospital perspective. Little or nothing is known about the costs of surgeries at the district hospitals (DHs) and the financial burden of surgery on these facilities on which the majority of rural populations rely.

 A total of 367 facilities provide major surgery services in Tanzania.^15^ The DHs (85 in all) provide first-level major surgical services for patients referred from lower-level facilities (4249 dispensaries, 586 health centres). Some of the latter (72 dispensaries and 104 health centres) have been upgraded to provide basic emergency SOA care.^15^ Surgeries at DHs are performed by medical officers (MOs) and assistant medical officers (AMOs), who are non-physician clinicians (NPCs) with advanced diplomas in medicine and surgery. Both cadres have undergone limited, non-specialist training in SOA care. DHs refer patients that they cannot manage primarily to regional referral hospitals (RHs).

 Tanzania lags behind in the LCGS indices, with staff shortages identified as the single most pressing deficit.^15^ Even though over 70% of the population lives in a rural area, most DHs do not have permanent SOA specialists, with lack of physician anaesthesiologists reported even up to the level of regional hospitals.^15,16^ In 2018, the country adopted its first NSOAP^15^ in line with the recommendation of the LCGS. It provides a systems-based pivot for all stakeholders towards a nationwide scale-up of safe surgery. The NSOAP clearly defines what surgeries are expected to be performed at every level of the health system. For instance, while it recommends non-operative fracture treatments at the DHs, the regional hospitals are expected to offer comprehensive elective and emergency orthopaedic services including ORIF.^15^

 SURG-Africa, which is a European Union (EU)-funded research project, has been implementing and evaluating a surgical mentorship model whereby specialists from higher-level hospitals provide mentorship and supervision support to surgical teams at DHs.^17^ This study in particular aimed to establish and compare the costs of performing common basic surgeries at DHs and a regional RH, taking into account both direct and indirect costs to the provider.

## Methods

###  Scope and Sample Selection

 The study was descriptive and cross-sectional, and took the perspective of the healthcare system. The period of interest was the 2017/2018 financial year (ie, July 1, 2017 to 30 June, 2018). Retrospective data collection took place from July to September 2018.

 Two DHs in Arusha region of Tanzania were selected based on the volume of surgery performed (as reported in the SURG-Africa situation analysis) and considerations of convenience: Meru DH and Oltrumet DH. In addition, we included their main RH: Mount Meru Regional Hospital (Mt Meru RH) in Arusha town.

###  Costing Tool 

 CostIt tool was used for data aggregation and analysis.^18^ This is an Excel-based tool for step-down accounting developed by the WHO-CHOICE (World Health Organization-CHOosing Interventions that are Cost-effective) initiative to help countries set their healthcare priorities. It is particularly recommended for costing of health services in LMICs. CostIt consists of a series of linked template worksheets that allow recording and analysis of cost data. We developed a data collection approach to mirror the data requirements of CostIt.

###  Data Collection and Organisation

####  Service Output Data

 Data on surgeries performed and other services were obtained from registers (theatre and ward) and aggregated Health Management Information System reports, using predesigned templates. Types of major surgeries of which at least 10 were performed at the RH during the 12-month study period and at least 5 at the DHs were selected for investigation. All other major and minor surgeries were grouped. At the RH, the other major surgeries were broken down by their respective subspecialties. Minor surgeries performed outside the main operating theatres (OT), ie, in minor theatres, out-patient department (OPD) or the casualty department, were excluded in the analysis. Classification of cases into “major” and “minor” was based on local practices as evidenced in the OT registers. It is noteworthy that the classifications were uniform across all the hospitals and essentially agreed with the common literature definition of a ‘major’ type of surgery: Any procedure occurring in a hospital OT, involving incision, excision, manipulation, or suturing of tissues, and usually under general or regional anaesthesia or sedation.^19^

###  Cost Data 

 Both economic and financial cost data were collected but only economic costs are reported in this paper. Hospital departments were divided into direct/clinical cost centres that deal with patients (eg, out-patient department, theatre, wards) and indirect/overhead cost centres that do not directly deal with patients but whose services are used by clinical cost centres. The sets of cost centres used in the analyses of the hospitals differed depending on their contexts. Details of these are presented in Tables S1-S3 (See Supplementary file 1). The cost burden of each cost centre was divided into capital costs (buildings, furniture, equipment, and vehicles) and recurrentcosts (salaries, allowances, consumables/supplies, and other running costs).

####  Capital Costs

 Economic capital costs were based on the replacement (current) costs, while financial costs were based on the historical (purchasing) costs.^20^ At Mt Meru RH, the full list of all the assets and their replacement costs, ages, assumed useful life (in years), and estimated remaining life were obtained from the hospital asset register. The asset valuation exercise had been conducted in 2016 by a professional valuing firm. At the DHs, there were no hospital asset registers, and therefore inventories of all available assets were established during data collection. In both cases, replacement costs of the items (equipment and furniture) were obtained from a combination of sources: Mt Meru RH asset register (on the assumption that similar items have the same costs across all facilities), procurement records, Medical Stores Department (MSD) annual price list, expert opinions (eg, procurement officers, transport officers), and online price quotations by local vendors.

 Historical costs of some of the buildings at Oltrumet DH were obtained from the managers. For these we calculated the average cost per m^2^. The costs of other buildings across the DHs were estimated by measuring their roof surface areas with Map Developers online tool – Area Calculator^21^ - and multiplying them by the average cost per m^2^.

 Conversions between historical and replacement costs were done by applying the appropriate World Bank gross domestic product (GDP) price deflators. In the absence of actual age data, the average ages from the Mt Meru RH asset register were used. Useful life was set at 66 years, 5 years and 10 years for buildings, vehicles and other assets (equipment/furniture), respectively. These numbers had been applied in the Mt Meru RH asset register.

 Calculation of the economic and financial capital costs is an automated process on the CostIt tool. Once the historical and replacement costs and the useful lives are entered, CostIt calculates the financial capital costs using a simple linear method and the economic capital costs using macro-generated annualization factors.^20^

####  Recurrent Costs

 For recurrent costs, we assumed that the economic costs are equal to the financial costs.^22^ Due to privacy concerns we were given the de-identified payroll records across the 3 hospitals. From this we calculated the average salary per level, per cadre. As the payroll records were not regularly updated with staff turnover, we obtained additional staff distribution and composition data from the heads of various departments and staff duty rosters. We multiplied the average salaries per cadre/level by the actual staff composition to arrive at a salary cost for each department. Distribution of salaries of clinicians over clinic (consultation), ward, OT and administrative (where applicable) duties were based on interviews of the clinicians. Costs of supplies to the various departments were estimated by multiplying the volumes used by the corresponding unit prices. Volumes of medical and non-medical supplies were obtained from the procurement office and pharmacy, respectively, using the supplies (requisition) records and ordering books. Unit prices for supplies were obtained from the procurement records and MSD official price lists. Other recurrent expenditures were obtained from the hospital annual financial reports.

 An overview of the cost items and their sources and calculations are presented in Table 1.

**Table 1:** Cost Items, Sources and Calculations

**Cost Items**	**Data Sources and Calculations**		
	**Mt Meru RH**	**Meru DH**	**Oltrumet DH**
**Capital Costs**			
Building sizes	Hospital asset register	Roof sizes measured with Map Developers online tool (Area Calculator)	Roof sizes measured with Map Developers online tool (Area Calculator)
Building historical costs	Hospital asset register	By multiplying total building roof area by per *m*^2^ cost of Oltrumet building costs	Costs of some buildings obtained from hospital managers; Costs of others obtained by multiplying the roof area by per *m*^2^ cost of the buildings with known costs
List of other assets (equipment, furniture, vehicles)	Hospital asset register	Hospital inventory taken by authors with specially designed inventory forms	Hospital inventory obtained from hospital managers
Costs of others assets (equipment, furniture, vehicles)	Hospital asset register	Mt Meru RH asset register; MSD official price list; procurement records; expert estimates; online price quotations by local vendors	Mt Meru RH asset register; MSD official list; procurement records; expert estimates; online price quotations by local vendors
Conversion between purchasing and replacement costs	Use of World Bank GDP deflator factors	Use of World Bank GDP deflator factors	Use of World Bank GDP deflator factors
Capital (depreciation) costs	Automated on CostIt tool^21^	Automated on CostIt tool^21^	Automated on CostIt tool^21^
** Recurrent Costs**			
Actual staff distribution/composition	Heads of departments and duty rosters	Heads of departments and duty rosters	Heads of departments and duty rosters
Average salary rates per cadre and per level	Calculated from de-identified payroll records	Calculated from de-identified payroll records	Calculated from de-identified payroll records
Salary load per department	Multiplication of the average salary rates and the staff composition data	Multiplication of the average salary rates and the staff composition data	Multiplication of the average salary rates and the staff composition data
Volumes of non-medical supplies to different departments	Procurement department records	Procurement department records/departmental order books	Matron’s supplies record
Volumes of drugs/medical supplies	Pharmacy department	Pharmacy department/departmental order books	Pharmacy records
Unit prices of supplies	MSD official price list; procurement records; expert estimates	MSD official price list; procurement records; expert estimates	MSD official price list; procurement records; expert estimates
Costs of non-medical supplies per department	Unit prices multiplied by the volume of supplies	Unit prices multiplied by the volume of supplies	Unit prices multiplied by the volume of supplies
Costs of drugs/medical supplies per department	Unit prices multiplied by the volume of supplies	Unit prices multiplied by the volume of supplies	Unit prices multiplied by the volume of supplies
Other recurrent costs (running costs, administrative costs, utilities etc)	Annual hospital financial report	Annual hospital financial report	Annual hospital financial report

Abbreviations: DH, district hospital; RH, referral hospital; MSD, Medical Stores Department; GDP, gross domestic product.

###  Costing Methodologies 

 After collecting the direct costs for each overhead and clinical cost centre, a combination of (top-down) step-down costing (SDC) and (bottom-up) activity-based costing (ABC) approaches was used to estimate the unit cost per surgical procedure. SDC was used to allocate costs from overhead cost centres to clinical cost centres. ABC was used to allocate costs from clinical cost centres to individual surgical procedures. A discount rate of 3% was used.^20^

####  Step-Down Costing 

 Allocation criteria were determined together with staff members. Criteria were chosen based on relevance (ie, how well they capture the main cost-incurring activities of a department) and the availability of data. Commonly used allocation criteria were direct personnel cost, number of nurses, and economic costs of buildings. More details about the allocation criteria used can be found in Tables S1-S3 (See Supplementary file 1).

####  Activity-Based Costing 

 Not all clinical departments contribute substantially to surgery. In the absence of sufficiently discriminatory records, the share of the total cost of a department pertaining to surgeries was estimated by the surgical proportion of all the patients it served ie, based on simple patient counts (as obtained from ward registers, theatre registers, and Health Management Information System records). This ranged from 0% for departments that have little or no contact with surgery patients (eg, medical wards) to 100% for departments that provide surgical services exclusively (eg, OT). This proportion was multiplied by its total cost (including overhead costs allocated to it in the SDC) to arrive at the total cost of surgery for the department. Only patients who were treated operatively were considered in this allocation formula. For instance, the fraction of total maternity ward cost allocated to surgery was equal to the ratio of all major obstetrics and gynaecological surgeries recorded in the theatres to the total number of patients admitted in the maternity wards during the study period. This approach assumes that both surgical and non-surgical patients consume resources equally.

 ABC was employed to establish the unit costs of the different types of surgery from the total costs of the different departments while reflecting the heterogeneity in the resource consumptions of the different surgeries. ‘Activities’ represent those areas in which different surgeries can realistically be expected to differ with regard to resource use and costs. They correspond to the domains in the surgical care pathway. The identified activities were: number of preoperative consultations, investigative activities (laboratory and imaging; diagnostic and routine surgical fitness assessment tests), pre-operative ward days, time spent in OT, post-operative ward days, and number of follow-up consultations. Post-operative investigations were considered exceptions and not norms, and therefore excluded. We assumed that resource utilization per unit of a given activity (eg, per day in the ward, per minute in the theatre or per consultation visit) is constant, irrespective of the type of surgery. While intra-operative resource use would expectedly differ across different surgeries, OT duration was used as a proxy, assuming that longer surgeries would logically consume more resources (such as anaesthetics, analgesics, intravenous fluids, antibiotics and sutures).

 For each type of surgery under our consideration, we estimated its “activity indices” by interviewing surgery clinicians (specialists, MOs, and AMOs) on their ‘standard practices’ (ie, patient management course from the first to the last contact). Such an approach had been applied in previous studies.^9,23^ Each clinician was interviewed on specific procedures he/she performed most commonly. For a procedure performed by more than one clinician in a hospital, we obtained multiple activity estimates and used the arithmetic means in the analyses. As more types of surgery were done than the common basic ones under our consideration we had to make assumptions regarding the activities of the other types of surgery, as they also consumed resources meant for surgery. For these we took the average activity indices across other surgeries in the respective sub-specialties.

 Each activity constituted a discrete cost pool. The costs of surgery per clinical department were distributed over the corresponding activity cost pools. For instance, the cost of OT was assigned wholly to the intra-operative activity cost pool while ward costs were split between pre-operative and post-operative care cost pools. Each activity cost pool was subsequently allocated to individual types of surgery based on the estimated activity indices. By summing up all costs allocated to a type of surgery in this way we obtained a total cost per type of surgery. We arrived at a unit cost for a particular procedure by dividing this total cost by the number of surgeries of that type performed.

 Costs are reported in December 2017 United States Dollars, ie, at the mid-point of the study period.

###  Sensitivity Analysis

 In order to understand how the uncertainties around our assumptions and input variables affect the results, we performed the following secondary analyses:

 1. Univariate Deterministic Sensitivity Analysis:We changed our assumptions and input variables one at a time (keeping other factors constant). The following variables were adjusted:

- Step-down allocation factors: Five selected allocation factors were substituted in each hospital. The affected factors are presented in Tables S1-S3 (See Supplementary file 1).
- Assumptions about allocation of departmental costs to surgery pool: The secondary model assumed that surgical patients consume twice as much resources as the non-surgical patients.
- Assuming the input costs were increased or decreased by 20%: Input costs adjusted were; (*i*) Assets (buildings, equipment, furniture, vehicles), (*ii*) Salaries, and (*iii*) Supplies.
- Useful life assumed for buildings was changed to 30 years, in line with WHO hospital cost analysis manual.^20^

2. Rather than proceeding with ABC, the total costs of surgeries per hospital were divided by the respective total numbers of surgeries performed in the theatres, thus giving single overall average costs of surgeries per hospital.

 For each procedure, a range of costs was presented based on the lowest and highest cost results from the univariate sensitivity analysis.

## Results

###  Study Hospital Descriptions

Table 2 presents the key parameters of the study hospitals with regards to infrastructure, human resource, and service delivery. The status of Mt Meru as the regional RH is reflected in it having more ward capacity (379 beds) than the DHs (110 and 63 for Meru and Oltrumet, respectively). Regarding infrastructure for surgery, it was the only hospital among the 3 with dedicated surgical wards and a specialized obstetrics/gynaecology theatre. Each of the 3 hospitals had a general theatre complex with 2 major operating rooms as well as a minor theatre. Mt Meru RH employed 7 specialist surgeons, along with 4 MOs and 2 AMOs performing surgeries. At Meru DH surgeries were done by 11 MOs and 8 AMOs, whereas Oltrumet DH had 2 MOs and 3 AMO’s performing surgeries.

**Table 2:** Relevant Surgical Parameters of the Study Hospitals

	**Mt Meru RH**	**Meru DH**	**Oltrumet DH**
**Infrastructure**			
Wards	13	5	3
ICU	1	-	-
Hospital beds	379	110	66
*… of which surgical beds*	94	-	-
Major theatre rooms	2	2	2
Specialized theatres	1	-	-
Minor theatres	1	1	1
Laundry machines	1	1	-
Anaesthetic machines	4	2	-
Sterilization machines (in the major theatre)	1	1	1
**Staffing**			
Total number of employed personnel (regular staff)	446	204	95
Surgeons	5	-	-
*… of which general surgeons*	2	-	-
*… Orthopaedic surgeon*	1	-	-
*… Otorhinolaryngologist*	1	-	-
*… Maxillofacial surgeon*	1	-	-
*... involved in general administration*	1	-	-
Obstetricians and gynaecologists	2	-	-
Anaesthesiologists	-	-	-
Physicians	3	-	-
... *of which radiologists*	1	-	-
General medical doctors	16	11	8
*… of which performing major surgeries*	4	11	2
*... involved in general administration*	-	1	1
General dental officers	2	2	1
AMOs	11	8	5
*… of which performing major surgeries*	2	8	3
*... trained in anaesthesia*	1	-	-
*... trained in radiology *	1	1	-
COs	3	12	13
Nurses (including nurse attendants)	229	119	28
... *of which trained in anaesthesia*	4	2	1
Other regular staff members	175	54	40
Service Output			
Admissions	20 557	7490	3963
Major surgeries performed in the major theatres	3457	843	390
*… of which caesarean sections *	2374 (69%)	767 (91%)	367 (94%)
Minor surgeries performed in the major theatres	1013	8	7

Abbreviations: ICU, intensive care unit; AMOs, assistant medical officers; COs, clinical officers; DH, district hospital; RH, referral hospital.

 The number of admissions was about 21 000 at Mt Meru RH, compared to less than 4000 at the DHs. Mt Meru RH performed 3457 major surgeries, compared to 843 at Meru DH and 367 at Oltrumet DH. These represented 17% of all admissions at the RH and 10.5% at the DHs. The majority of surgeries performed at all hospitals were CSs, accounting for over 90% at the DHs. As expected of a RH, Mt Meru had a relatively wider range of cases, with CS accounting for 69%. Non-CS cases at the RH included 29 cases of ORIF compared to 100 cases of surgical debridement (plus conservative treatment). Details of the surgeries and their volumes are presented in Table S4 (See Supplementary file 1).

###  Costs of Running the Hospitals 

 The total direct costs of overhead and clinical departments of Mt Meru RH were $907k and $2616k, respectively. At Meru DH, the overhead and clinical centres incurred total direct costs of $294k and $1247k respectively. Overhead and clinical departments of Oltrumet costs were $139k and $443k respectively. Although the absolute figures differed among the hospitals as a function of their size, several patterns could be seen. Salaries took up the largest share of the costs. Of the overhead departments, the general administration department was the most costly, owing in part to the fact that certain overhead costs could not be allocated to particular direct cost centres. At Mt Meru, the pharmacy department was also relatively costly due to the large size of the building it occupies and the relatively large number of academically trained personnel in the department. The direct costs of the clinical departments were much higher than those of most overhead departments, owing mostly to their higher personnel counts. Across all the hospitals, the OPD and wards were generally the most costly departments, followed by diagnostic departments and the OT. Details of these costs are shown in Tables S5-S10 (See Supplementary file 1).

 Following the step-down of overhead costs, the total economic costs of the clinical departments for the 12 month period were: $3453k for Mt Meru RH, $1543k for Meru DH, and $576k for Oltrumet DH. The relative size of the capital costs at Mt Meru RH was lower than that of the DHs. The details are presented in Tables S11-S13 (See Supplementary file 1).

###  Total Costs of Surgeries 

Tables 3-5 present the details of the allocations of the different clinical departments to surgery. Even though Mt Meru RH had a surgical ward and a surgical OPD which were 100% dedicated to surgery patients, the costs absorbed in determining the unit costs of surgeries were only 54% and 60% respectively. The excluded costs pertain to the patients in those departments who were treated non-operatively. Maternity ward and Reproductive and Child Health department allocated 18% of their costs (each) to surgery services at all 3 facilities. At the DHs where the wards had mixed patients, surgery patients accounted for not more than 2% of costs in all the other cost centres.

**Table 3:** Summary of the Contributions of the Clinical Departments to Surgery Care at Mt Meru RH

**Cost Centres**	**Percentages Allocated to Surgery**	**Costs Allocated to Surgery (Thousands of USD)**
OPD/Casualty	Nil	0
Laboratory	7%	22
Radiology	7%	9
OG Theatre	100%	204
Main OT	100%	239
Paediatric ward	Nil	0
Maternity wards + Gynae OPD	18%	82
SOPD	60%	20
Eye department	Nil	0
Medical ward + CTC + TB unit	Nil	0
Surgical ward	54%	238
Dental Health	Nil	0
Physiotherapy + Mental Health + ICU	0%	0
**Total**		**813**

Abbreviations: RH, referral hospital; ICU, intensive care unit; CTC, care and treatment centre; TB, tuberculosis; OG, obstetrics and gynaecology; OT, operating theatres; OPD, out-patient department; SOPD, surgical OPD.

**Table 4:** Summary of the Contributions of the Clinical Departments to Surgery Care at Meru DH

**Cost Centres**	**Percentages Allocated to Surgery**	**Costs Allocated to Surgery (Thousands of USD)**
OPD (GOPD, POPD, BIMA and DM OPD)	1%	5
Laboratory	2%	2
Radiology	2%	1
Main operating theatre	100%	113
Minor operating theatre	Nil	0
Paediatric ward	0.30%	0
Maternity ward	18%	36
Female ward	1%	1
Male ward	2%	2
RCH (including CECAP unit)	18%	17
Physiotherapy + Nutrition + Health office	Nil	0
CTC + TB/Leprosy unit	Nil	0
Eye + Dental units	Nil	0
**Total**		**177**

Abbreviations: DH, district hospital; RCH, Reproductive and Child Health; OPD, out-patient department; GOPD, general OPD; POPD, paediatric OPD; BIMA, National Health Insurance Fund; DM, diabetes mellitus; CECAP, Cervical Cancer Prevention; CTC, Care and Treatment Centre; TB, tuberculosis.

**Table 5:** Summary of the Contributions of the Clinical Departments to Surgery Care at Oltrumet DH

**Cost Centres**	**Percentages Allocated to Surgery**	**Costs Allocated to Surgery (Thousands of USD)**
OPD/casualty	0.10%	0.1
Laboratory	2%	1
Radiology	2%	0
RCH cost	17%	6
Main operating theatre	100%	54
Minor operating theatre	Nil	0
Maternity ward	17%	17
Female (+paediatric) ward	0.50%	0
Male ward	2%	1
Physiotherapy	Nil	0
CTC + TB unit	Nil	0
Mental health	Nil	0
Dental unit	Nil	0
**Total**		**79**

Abbreviations: DH, district hospital; OPD, out-patient department; RCH, Reproductive and Child Health; CTC, Care and Treatment Centre; TB, tuberculosis.

**Figure 1:**
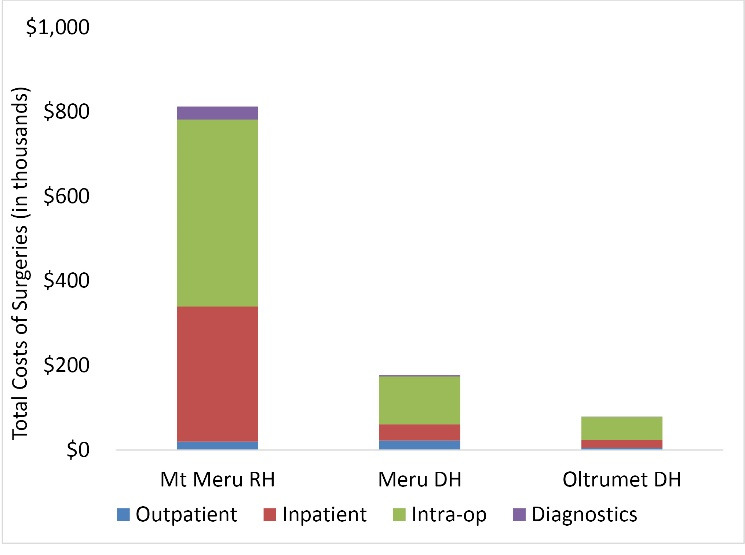
Total Costs of Surgeries, Disaggregated Into the Domains of Surgery Care. Abbreviations: DH, district hospital; RH, referral hospital.

Figure 1 presents the total cost of surgery per hospital, broken down by the domains in the surgical care pathway – diagnostics, intraoperative care, inpatient care, outpatient care. Mt Meru RH, Meru DH, and Oltrumet DH, spent $813k, $177k and $79k, respectively, on surgery care. At all the hospitals the highest proportion of the costs (at least 50%) were incurred in the OT. These were followed by costs of inpatient care and then diagnostics and surgical OPD activities.

 Disaggregating the total costs of surgery into types of costs (recurrent versus capital), salaries constituted at least 70% of the total cost of surgery care at the 3 hospitals (not shown on the plot). Again, the relative share of capital costs was lowest (2%) at Mt Meru (the biggest hospital), 6% at Meru DH and highest (11%) at Oltrumet DH (the smallest hospital).

 Comparing the total costs of surgical services to the total hospital costs, Mt Meru RH spent over a fifth (22%) of its resources on surgical care while Meru DH and Oltrumet DH spent 12% and 14%, respectively.

###  Unit Costs of Surgeries

**Figure 2:**
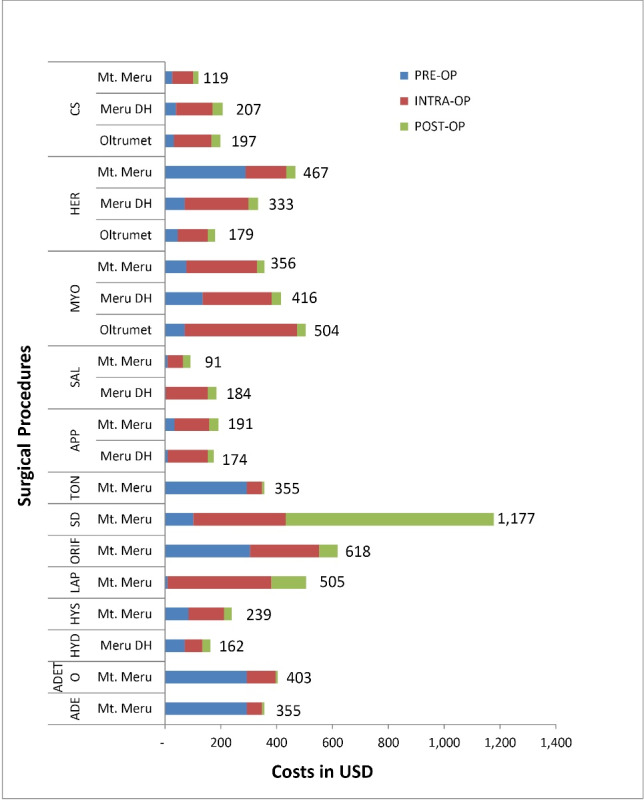
Units Costs of Surgeries, Disaggregated into Pre-operative, Intra-operative and Post-operative Stages. Abbreviations: DH, district hospital; ADE, adenectomy; ADETO, adenotonsillectomy; APP, appendectomy; CS, caesarean section; HER, herniorrhaphy; HYD, hydrocoelectomy; HYS, hysterectomy; LAP, laparotomy; MYO, myomectomy; ORIF, open reduction and internal fixation; SAL, salpingectomy; SD, surgical debridement; TON, tonsillectomy; PRE-OP, pre-operative; INTRA-OP, intra-operative; POST-OP, post-operative.

 Tables S14-S16 (See Supplementary file 1) show the estimates of the activity indices of the different surgery types, as obtained from interviews of the performing clinicians. Figure 2 shows the unit costs of the most commonly performed surgical procedures, with disaggregation into pre-operative, intra-operative, and post-operative stages of care. Asides herniorrhaphy which was most costly at Mt Meru RH, unit costs were lower at the RH than the DHs. While a herniorrhaphy costs $179 at Oltrumet DH, it costs $333 at Meru DH and $467 at Mt Meru RH. A CS cost $119 at Mt Meru RH and nearly twice as much at Meru DH ($207) and Oltrumet DH ($197). Myomectomy, which was most expensive at Oltrumet DH ($504) costs $416 at Meru DH and $356 at the RH. Appendectomy costs were comparable between Mt Meru RH ($191) and Meru DH ($174) while salpingectomy performed at Meru DH ($184) cost twice as much as that at Mt Meru RH ($91). Overall, salpingectomy at the RH stood out as the cheapest of all the procedures. On the other hand, surgical debridement at Mt Meru RH ($1177) was the most expensive, followed by ORIF ($618).

 With regards to costs in different stages of care, about 64% of the procedures incurred at least 60% of their costs intraoperatively. At Mt Meru RH, surgical debridement (plus conservative treatment) stood out with high post-operative costs due to prolonged inward care of at least 4 weeks, but with low pre-operative cost, being an emergency operation. On the other hand, ORIF (mostly an elective procedure in this setting), with extended pre-operative consultation visits (up to 4), had a higher pre-operative cost. Ear-nose-throat procedures (tonsillectomies/adenectomies/adenotonsillectomies) were unique in that large percentages (78% on average) of their costs were incurred pre-operatively. This was due to their short OT times of 20–45 minutes and post-operative care of less than 24 hours.

 The results of the sensitivity analyses are presented in Table 6. Change in criteria of allocating costs of different departments to surgery had the largest effect on the results (about 11%–53% increase in the unit costs), followed by changes in costs of salaries (about 15% change). Accordingly, results of the scenario of 20% decrease in salaries formed the lower margin of the costs ranges. The ranges of costs of CSs for instance were US$103–150, US$176–263, and US$170–242 at Mt Meru RH, Meru DH and Oltrumet DH, respectively. Bypassing the ABC, the overall average unit costs of surgeries were US$182, US$208 and US$199 at Mt Meru RH, Meru DH and Oltrumet DH, respectively.

**Table 6:** Results of Sensitivity Analyses

**Procedures**	**Hospital **	**Base Costs (USD)**	**Changes in Step-Down Allocation Criteria**	**Change in Estimated Useful Life**	**Assets Costs**		**Salaries Costs **		**Supplies Costs**		**Assuming Surgical Patients Consume Resource Twice as Much as Non-surgical Patients**	**Ranges **	**Total Costs of Surgeries Divided by Total Number of Surgeries**
					**+20%**	**-20%**	**+20%**	**-20%**	**+20%**	**-20%**			
CS	Mt Meru RH	119	116 (-3%)	120 (+1%)	120 (+0%)	119 (-0%)	136 (+14%)	103 (-14%)	126 (+6%)	112 (-6%)	150 (+26%)	103-150	182
	Meru DH	207	209 (+1%)	208 (+1%)	210 (+2%)	203 (-2%)	237 (+15%)	176 (-15%)	214 (+4%)	199 (-4%)	263 (+28%)	176-263	208
	Oltrumet DH	197	220 (11%)	202 (+2%)	202 (+2%)	193 (-2%)	225 (+14%)	170 (-14%)	204 (+4%)	190 (-4%)	242 (23%)	170-242	199
HER	Mt Meru RH	467	467 (+0%)	468 (+0%)	469 (+1%)	464 (-1%)	536 (+15%)	397 (-15%)	489 (+5%)	445 (-5%)	592 (+27%)	397-592	182
	Meru DH	333	339 (+2%)	336 (+1%)	339 (+2%)	328 (-2%)	383 (+15%)	283 (-15%)	345 (+3%)	322 (-3%)	426 (+28%)	283-426	208
	Oltrumet DH	179	198 (10%)	184 (+2 %)	184 (+2%)	175 (-2%)	205 (+14%)	154 (-14%)	186 (+4%)	173 (-4%)	243 (36%)	154-243	199
MYO	Mt Meru RH	356	344 (-3%)	359 (+0%)	358 (+0%)	355 (-0%)	406 (+14%)	307 (-14%)	377 (+5%)	336 (-6%)	428 (+20%)	307-428	182
	Meru DH	416	420 (+1%)	419 (+1%)	422 (+2%)	409 (-2%)	478 (+15%)	353 (-15%)	430 (+3%)	401 (-3%)	552 (33%)	353-552	208
	Oltrumet DH	504	574 (14%)	518 (+3%)	517 (+3%)	491 (-3%)	573 (+14%)	435 (-14%)	522 (+4%)	486 (-4%)	590 (17%)	435-590	199
SAL	Mt Meru RH	91	89 (-2%)	92 (+1%)	92 (+0%)	91 (-0%)	104 (+14%)	79 (-14%)	97 (+6%)	86 (-6%)	118 (+29%)	79-118	182
	Meru DH	184	188 (+2%)	185 (+1%)	187 (+2%)	180 (-2%)	211 (+15%)	157 (-15%)	190 (+4%)	177 (-4%)	214 (+17%)	157-214	208
APP	Mt Meru RH	191	189 (-1%)	192 (+1%)	192 (+0%)	190 (-0%)	219 (+15%)	162 (-15%)	200 (+5%)	182 (-5%)	219 (+15%)	162-219	182
	Meru DH	174	178 (+2%)	175 (+1%)	177 (+2%)	171 (-2%)	219 (+15%)	149 (-15%)	180 (+4%)	168 (-4%)	199 (+15%)	149-199	208
TON	Mt Meru RH	355	358 (+1%)	356 (+0%)	357 (+1%)	353 (-1%)	408 (+15%)	303 (-15%)	372 (+5%)	338 (-5%)	471 (+33%)	303-471	182
SD	Mt Meru RH	1177	1179 (+0%)	1182 (+0%)	1183 (+1%)	1172 (-1%)	1352 (+15%)	1003 (-15%)	1233 (+4%)	1122 (-5%)	1509 (+28%)	1003-1509	182
ORIF	Mt Meru RH	618	617 (-0%)	620 (+0%)	621 (+0%)	615 (-0%)	710 (+15%)	525 (-15%)	646 (+4%)	589 (-5%)	757 (+23%)	525-757	182
LAP	Mt Meru RH	505	497 (-2%)	508 (+0%)	507 (+0%)	503 (-0%)	581 (+15%)	429 (-15%)	528 (+5%)	482 (-5%)	560 (+11%)	429-560	182
HYS	Mt Meru RH	239	236 (-1%)	240 (+0%)	240 (+0%)	238 (-0%)	272 (+15%)	205 (-14%)	252 (+6%)	225 (-6%)	310 (+30%)	205-310	182
HYD	Meru DH	162	162 (+0%)	163 (+1%)	164 (+1%)	160 (-1%)	187 (+16%)	137 (-16%)	167 (+3%)	157 (-3%)	249 (+53%)	137-249	208
ADETO	Mt Meru RH	403	405 (+0%)	405 (+0%)	405 (+1%)	401 (-1%)	463 (+15%)	344 (-15%)	422 (+5%)	382 (-5%)	519 (+29%)	344-519	182
ADE	Mt Meru RH	355	358 (+1%)	356 (+0%)	357 (+1%)	353 (-1%)	408 (+15%)	303 (-15%)	372 (+5%)	338 (-5%)	471 (+33%)	303-471	182

*All values have been rounded to the nearest whole numbers. Abbreviations: ADE, adenectomy; ADETO, adenotonsillectomy; APP, appendectomy; CS, caesarean section; HER, herniorrhaphy; HYD, hydrocoelectomy; HYS, hysterectomy; LAP, laparotomy; MYO, myomectomy; ORIF, open reduction and internal fixation; SAL, salpingectomy; SD, surgical debridement; TON, tonsillectomy; DH, district hospital; RH, referral hospital.

## Discussion

 Applying a combination of SDC and ABC, this study quantified the costs of performing common surgeries in a regional hospital and 2 DHs in Arusha region of Tanzania. It also gives insights into the costs of running the hospitals. As the Government of Tanzania implements its first NSOAP, this study therefore provides vital evidence for planning and budgeting of surgery scale-up at the regional and district facility levels.

 Unit costs and the relative shares of capital costs were generally lower at the RH. This reflects advantages of economies of scale and scope of surgery at the RH, and conversely, a possible underutilisation of capacities at the DHs. The total cost of running the clinical departments of Mt Meru RH (after overhead step-down) was 2 times and 6 times those of Meru DH and Oltrumet DH, respectively. However, the volume of surgery output was about 4 times and 9 times those of Meru DH and Oltrumet DH, respectively. Similarly, with 3 major operating rooms, Mt Meru RH performed nearly 3500 major surgeries over the one-year study period, giving an average of almost 1200 per theatre. In contrast, Meru and Oltrumet DHs, with 2 major operating rooms each, performed an average of about 400 and less than 200 per theatre, respectively.

 From a provider perspective, it makes economic sense, especially in resource poor settings, to concentrate resources and surgery services at RHs for reasons of efficiency and reduced health system costs. However, such policies often come with consequences of increased access distance and costs of services on the patients, or outright lack thereof – potentially compromising health system equity objectives.^24^ While this study did not evaluate demand side costs, there is ample evidence that patients incur substantial costs in seeking care at the higher hospitals which are often far from their homes; driven by transport, feeding, lodging and informal care.^25-27^ Moreover, judging by the NSOAP stipulations,^15^ the bulk of surgeries at the RH (eg, appendectomies, herniorrhaphies, and hydrocoelectomies) are ideally provided at the DHs, and these could crowd out the more advanced RH-appropriate cases. A scale-up of the DH-level appropriate surgeries will not only drive down unit costs at this level but also allow the RHs to concentrate on and scale up the more complex and resource intensive cases,^28^ thus allowing even a more judicious application of the RH resources.

 Our findings do not only support scale-up of DH-appropriate surgery, but also provide insights into how this could be achieved. Firstly, in discussions about surgery scale-up in LMICs, there is a tendency to prioritise extra investments in physical infrastructure expansion.^29^ While this is relevant, we contend that in many instances, as is seen in this study and elsewhere,^7^ there are trapped potentials at the DH that require only minimal input to be enabled. Efforts must be made to first identify and tackle such rather intangible, rate-limiting elements inhibiting full exploitation of the already installed (capital and human) capacities at the DHs. These include the supplies, instruments, and staff mix. The absence of one anaesthetist for instance could render a battalion of doctors redundant.^16^

 Secondly, having large numbers of clinicians at the DHs without the requisite skills induces inefficiencies. While the DHs employed several AMOs/MOs (18 at Meru, 11 at Oltrumet), only very few of them performed surgeries other than emergency CS cases encountered during on-call hours; such that less than 10% of all the surgeries at the DHs were non-CS. An earlier study in Tanzania reported that over 70% of the patients seen at the national hospital were self-referred, bypassing the DHs; nearly 70% were surgery cases; and as much as 96% cited lack of expertise at DH as the reason for self-referrals.^30^ Among those formally referred from the lower centres, lack of expertise alongside equipment was again the most commonly cited reason for referrals.^30^ To derive full benefits from the manpower available at the DHs, disincentives for self-referrals to RHs (such as introduction and enforcement of penalty fees), and a mechanism of continuing surgical training and supervision at the DHs (such as trialled in SURG-Africa) will be needed.^17^

 Thirdly, salaries accounted for at least 70% of the entire costs of surgical care across the 3 hospitals. Although similar findings have been reported in the past (eg, Kruk et al),^23^ a study done in Malawi found the share of salaries to be significantly lower (about 31% in the theatre).^7^ Malawi public service salaries are generally lower than Tanzania salaries,^31^ and surgeries at DHSs are provided mostly by clinical officers (COs)^32^ contrary to the practice in Tanzania, where surgeries at the DHs are performed by MOs and AMOs. As human resources constitute the bulk of costs of surgery, our findings suggest that from an economic point of view, task shifting is a wise and pragmatic approach (at least in the medium term) to providing surgery to rural populations in the face of severe manpower shortages. While there have been safety concerns about surgeries performed by NPCs,^33^ and there have been no randomized controlled trials comparing surgeries performed by medical doctors and NPCs,^34^ it is interesting to note that several controlled studies in Tanzania^35,36^ and elsewhere^37,38^ have reported absence of statistically significant differences in health outcomes.

 Lastly, this study makes an economic case for the provision of implants at the RH. Most open fractures at the RH were managed by surgical debridement and conservative treatments such as casting, slabbing, and traction (or external fixation).^39,40^ These entailed several weeks of post-operative inpatient care (constituting 63% of the total costs), compared to few days for ORIF patients. Granted that increased risk of infection with use of internal fixation as a first line treatment in open fractures may be one reason for this practice, the other major reason is unavailability of implants which are prohibitively expensive^39,40^; even closed fractures requiring surgical fixation are commonly managed conservatively for this reason.^14,41^ This study reveals that surgical debridement plus conservative treatment (US$1177) is in fact more costly than ORIF (US$618) as a result of this prolonged ward stay. Although the ORIF cost here excludes the cost of implants (about US$134 ^14^), since they were not captured in the routine hospital cost records, it includes the costs of prolonged pre-operative clinic visits. Indeed, cost-effectiveness studies in Kenya^42^ and the United States^43^ had demonstrated that internal fixation offers better clinical outcomes at lower costs compared to non-operative fixation of fractures. This illustrates the need for scaling up basic surgery at the DH-level, as this can free up resources at the RH that could then be channelled to such critical inputs as orthopaedic implants.

###  Strengths

 Our study draws strength from the use of (primary) hospital-level data, thus making the results more policy relevant. Further, the study joins the few published surgery costing studies in sub-Saharan Africa that delineate pre-, intra-, and post-operative costs.^8,9^ This approach gives a better insight into resource flows and utilisation thereby revealing the potentials for efficiency gains. This can guide hospital managers and clinicians in making more rational resource allocations as well as identifying necessary changes in clinical practices.

###  Limitations

 The main limitation of our study is that data on “standard practice” applied in the ABC were obtained through interviews of clinicians rather than observation of actual practice, leaving room for respondent biases in the estimates. Moreover, these estimates of activities for ‘typical’ cases do not capture atypical situations or scenarios with complications. Interviewing of multiple clinicians about the same type of surgery was an attempt to reduce the possible bias however. Further studies and complementary approaches, for example time and motion studies, are encouraged to obtain finer insights into the cost dynamics of providing surgeries in LMICs.

## Conclusion

 While there are economic reasons to concentrate surgeries at the bigger specialist hospitals, overconcentration of relatively simple surgeries at the RH leads to congestion at this level at the expense of specialist services. Evidence of underutilised capacities at the DHs provides justification and potentials for scaling up surgery at the DH-level. Further initiatives would need to prioritise the rate limiting factors at DHs, such as staff skills, anaesthesia capacity and supplies, over physical infrastructure extension.

## Acknowledgements

 We wish to acknowledge the support of the Ministry of Health, Community Development, Gender, Elderly and Children (MOHCDGEC) of Tanzania; the President’s Office, Regional Administration and Local Government (PORALG) of Tanzania; Tanzania Surgical Society (TSA); East, Central and Sothern African Health Community (ECSA-HC); and Arusha Regional Medical Office. We thank specially the management teams and staff members of the hospitals involved in the study for their assistance and cooperation.

## Ethical issues

 This study was approved by the Kilimanjaro Christian Medical College Research Ethics and Review Committee (approval no. CRERC 2026), and the National Institute for Medical Research of Tanzania (approval no. NIMR/HQ/R.8a/Vol. IX/2600). The costing studies required collection of data that may be considered sensitive; especially regarding financial records, staff grades, salary levels and staff time allocation. Verbal informed consents were obtained at each of the institutions involved from the management team members, relevant clinicians and all other respondents.

## Competing interests

 Authors declare that they have no competing interests.

## Authors’ contributions

 Conception and Design: MI, HB, LB. Acquisition of data: MI, AJ, HB, LB. Analysis and interpretation of data: MI, HB, LB. Drafting of Manuscript: MI, HB, LB. Critical revision of the manuscript for important intellectual content: All co-authors. Funding acquisition: JK, KC, EK, RB, LB. Administrative, technical, or material support: AJ, EK. Supervision: LB.

## Funding

 This study was part of the Scaling up Safe Surgery for District and Rural Populations in Africa (SURG-Africa) project, which is funded by the European Union’s Horizon 2020 Programme for Research and Innovation, under grant agreement no: 733391.

## Supplementary files

Supplementary file 1 contains Tables S1-S16.
