# Supplementary material for: Economic Costs of Providing District- and Regional-Level Surgeries in Tanzania
Source: Int J Health Policy Manag. 2021 Feb 23;11(7):1120–31. doi: 10.34172/ijhpm.2021.09 (PMC9808166; doi:10.34172/ijhpm.2021.09)
Supplement: Supplementary file 1 — contains Tables S1-S16. [file ijhpm-11-1120-s001.pdf]

Supplementary file 1

Table S1. Assumptions and Allocation Factors for Step-Down of Overhead Costs for Mt Meru RH

**Department**	**Allocation factors in base analysis**	**Assumptions/Special considerations**	**Allocation factors in secondary analysis**
General Administration	*Cost of personnel*		*Staff number*
Medical Stores	*Volume of supplies*		*Volume of supplies*
Medical Engineering	*Cost of the equipment*	*Only clinical departments were considered*	*Cost of the equipment*
General Transport	*Personnel cost*		*Number of Inpatient*
Kitchen	*Number of inpatients*		*Number of inpatients*
Laundry Services	*Number of nurses & MA's*		*Costs of buildings*
Environmentals + security	*Cost of the buildings*		*Personnel Cost*
Housing + Mortuary	*Cost of personnel*	*Only clinical departments were considered*	*Number of nurses/MA*
Medical Records Services (including ICT)	*Cost of personnel*		*Cost of personnel*
Pharmacy	*Number of nurses & MA's*	*Radiology and laboratory departments were excluded*	*Number of nurses & MA's*
Utilities	*Cost of the buildings*		*Cost of the buildings*

Table S2. Assumptions and Allocation Factors for Step-Down of Overhead Costs for Meru DH

**Department**	**Allocation factors in base analysis**	**Assumptions/Special considerations**	**Allocation factors in secondary analysis**
General Administration	*Cost of personnel*		*Staff number*
Medical Stores	*Volume of supplies*		*Volume of supplies*
Registry (Staff records) and M&E Unit	*Number of staff members*		*Cost of the equipment*
General Transport/DIVO	*Personnel cost*		*Number of Inpatient*
Kitchen + Housing	*Number of staff members*	*Kitchen here serves staff and not patients*	*Number of inpatients*
Laundry Services	*Number of nurses & MA's*	*Number of nurses correlate with patient load*	*Costs of buildings*

Social Welfare Department	*Number of nurses & MA's*		*Personnel Cost*
BIMA Records	*Number of nurses & MA's*		*Number of nurses/MA*
Medical Records Services	*Number of nurses & MA's*		*Cost of personnel*
Dispensary/Pharmacy	*Number of nurses & MA's*	*Physiology, radiology and laboratory departments were excluded*	*Number of nurses & MA's*
Utilities	*Cost of the buildings*		*Cost of the buildings*

Table S3. Assumptions and Allocation Factors for Step-Down of Overhead Costs for Oltrumet DH

Department	Allocation factors in base analysis	Assumptions/Special considerations	Allocation factors in secondary analysis
General Administration	*Cost of personnel*		*Number of Staff*
Accounts	*Cost of personnel*		*Number of Staff*
Staff Housing	*Number of staff members*		*Personnel cost*
General Transport	*Personnel cost*		*Personnel cost*
Kitchen	*Number of staff members*	*Kitchen here serves staff and not patients*	*Personnel cost*
Laundry Services	*Number of nurses & MA's*		*Cost of buildings*
Security	*Cost (size) of buildings*		*Cost (size) of buildings*
Mortuary	*Number of admissions*		*Number of admissions*
Medical Records Services	*Number of nurses & MA's*		*Number of nurses & MA's*
Dispensary/Pharmacy	*Number of nurses & MA's*	*Physiotherapy, radiology and laboratory depts were excluded*	*Number of nurses & MA's*
Utilities	*Cost of the buildings*		*Cost of the buildings*

Table S4. Types of Major Surgeries Performed at the Hospitals and Their Volumes

Types of surgeries	Volumes (number of procedures performed in 12 months)		
	Mt Meru RH	Meru DH	Oltrumet DH
Herniorrhaphy	163	14	8

CS	2,374	767	367
Myomectomy	13	10	5
Salpingectomy	102	23	-
Appendectomy	70	13	-
Laparotomy	177	-	-
Adenotonsillectomy	16	-	-
Tonsillectomy	21	-	
Adenectomy	31	-	-
Surgical debridement	100	-	-
ORIF	29	-	-
Hysterectomy	88	-	-
Hydrocoelectomy	-	5	-
Other major general surgeries	139	1	4
Other major Obs/Gynae surgeries	31	9	6
Other major orthopaedic surgeries	77	-	-
Other major ENT surgeries	26	-	-
**Total**	**3,457**	**842**	**390**

Table S5. Direct Costs of Overhead Departments at Mt Meru RH (in USD)

**COST CENTRES**	**Direct Costs**				
	Recurrent		Capital		Total
	Personnel	Other recurrent	Medical equipment	Other capital	
**General Administration**	228,159	20,295		4,385	259,839
**Medical Stores**	9,501	33,874		4,852	48,226
**Medical Engineering**	3,694	42,340		0	46,033

**General Transport**	17,448	0		34,162	51,610
**Kitchen**	15,085	16,867		2,466	34,419
**Laundry Services**	19,784	3,111		188	23,084
**Environmental Services + Security**	18,786	0		158	18,945
**Housing + Mortuary**	0	0		4,347	4,347
**Medical Records Services**	57,996	11,756		2,272	72,024
**Pharmacy**	69,710	819		165,298	235,827
**Utilities**	0	119,603		0	119,603
**Total**	440,163	248,664		218,128	906,955
	**49%**	**28%**	**0%**	**23%**	**100%**

Table S6. Direct Costs of Clinical Departments at Mt Meru RH (in USD)

**COST CENTRES**	**Direct Costs**				
	Recurrent		Capital		Total
	Personnel	Other recurrent	Medical equipment	Other capital	
**OPD/Casualty**	215,197	230,684		2,110	447,991
**Laboratory**	114,755	150,246	691	3,264	268,956
**Radiology**	62,566	11,793	6,047	1,596	82,002
**Obs/Gynae Theatre**	115,070	39,062	332	1,160	155,625
**Main Operating Theatre**	147,057	29,893	659	2,510	180,119
**Paediatric Ward**	218,173	5,559		11,514	235,245
**Maternity Wards + Gynae OPD**	232,996	47,938		16,235	297,169
**SOPD**	24,492	1,979		548	27,019
**Eye Department**	46,778	4,824		1,286	52,888
**Medical Ward + CTC + TB Unit**	290,842	12,683		17,564	321,089
**Surgical Ward**	261,420	60,302		10,776	332,498

**Dental Health**	49,499	6,997		543	57,039
**Physiotherapy + Mental Health + ICU**	147,529	6,650		4,478	158,657
**Total**	1,926,373	608,609	7,730	73,585	2,616,296
	**74%**	**23%**	**0.3%**	**3%**	**100%**

Table S7. Direct Costs of Overhead Departments at Meru DH (in USD)

COST CENTRES	Direct Costs				
	Recurrent		Capital		Total
	Personnel	Other recurrent	Medical equipment	Other capital	
**General Administration**	109,553	23,598		1,614	134,764
**Stores**	7,663	334		340	8,336
**Registry (Staff Records) and M&E Unit**	15,131	121		314	15,565
**General Transport/DIVO Unit**	12,041	12,574		25,746	50,361
**Kitchen + Housing**	0	0		4,929	4,929
**Laundry Services**	5,731	2,159		1,902	9,792
**Social Welfare Office**	5,747	640		350	6,736
**BIMA Records**	5,148	438		677	6,262
**Medical Record Service**	9,357	2,571		836	12,765
**Dispensary/Pharmacy**	34,321	624		1,664	36,609
**Utilities**	0	7,875		0	7,875
**Total**	204,691	50,932		38,371	293,994
	**73%**	**18%**	**0%**	**9%**	**100%**

Table S8. Direct Costs of Clinical Departments at Meru DH (in USD)

COST CENTRES	Direct Costs		
	Recurrent	Capital	Total

	Personnel	Other recurrent	Medical equipment	Other capital	
**OPD (GOPD, POPD, BIMA and DM OPD)**	170,574	178,775		5,544	354,893
**Laboratory**	71,000	6,860	8,750	905	87,515
**Radiology**	21,518	2,615	3,792	1,428	29,354
**Main Operating Theatre**	53,676	15,004	8,160	2,583	79,423
**Minor Operating Theatre**	54,919	14,067	641	1,184	70,811
**Paediatric Ward**	77,562	2,002		1,298	80,861
**Maternity Ward**	138,830	18,699		6,117	163,646
**Female Ward**	57,217	3,085		3,782	64,083
**Male Ward**	54,757	2,150		4,026	60,933
**RCH (Including CECAP Unit)**	64,259	1,049		6,530	71,838
**Physiotherapy + Nutrition + Health Office**	35,776	612		1,377	37,766
**CTC +TB/Leprosy Unit**	76,511	3,737		4,605	84,853
**Eye + Dental Units**	55,879	991		4,318	61,189
**Total**	932,479	249,646	21,343	43,697	1,247,165
	**75%**	**20%**	**2%**	**4%**	**100%**

Table S9. Direct Costs of Overhead Departments at Oltrumet DH (in USD)

**COST CENTRES**	**Direct Costs**				
	Recurrent		Capital		Total
	Personnel	Other recurrent	Medical equipment	Other capital	
**General Administration**	50,924	3,946		1,644	56,514
**Accounts**	3,877	33		345	4,255
**Staff Housing**	0.14	0		2,206	2,206
**General Transport**	1,174	7,756		28,123	37,053

**Kitchen**	0	0		294	294
**Laundry Service**	1,073	0		147	1,220
**Security**	3,218	0		0	3,218
**Mortuary**	3,910	0		294	4,204
**Medical Record Service**	9,482	4,205		309	13,996
**Dispensary/Pharmacy**	11,987	0		817	12,804
**Utilities**	0	3,464		0	3,464
**Total**	85,646	19,404		34,180	139,230
	**68%**	**15%**	**0%**	**17%**	**100**

Table S10. Direct Costs of Clinical Departments at Oltrumet DH (in USD)

**COST CENTRES**	**Direct Costs**				
	Recurrent		Capital		Total
	Personnel	Other recurrent	Medical equipment	Other capital	
**OPD (GOPD, POPD, BIMA and DM OPD)**	83,311	83,442		897	167,650
**Laboratory**	18,535	142	1,858	613	21,147
**Radiology**	3,763	80	388	242	4,473
**Main Operating Theatre**	21,710	235	1,250	806	24,001
**Minor Operating Theatre**	27,752	6,029	3,671	3,841	41,293
**Paediatric Ward**	8,653	1,507		57	10,217
**Maternity Ward**	61,196	8,839		4,333	74,368
**Female Ward**	21,645	13,652		4,025	39,322
**Male Ward**	15,321	2,884		2,241	20,447
**RCH (Including CECAP Unit)**	6,635	0		477	7,112
**Physiotherapy + Nutrition + Health Office**	16,039	28		872	16,939
**CTC +TB/Leprosy Unit**	5,192	0		238	5,430

**Eye + Dental Units**	8,694	87		1,892	10,673
**Total**	298,446	116,926	7,167	20,533	443,072
	**75%**	**20%**	**2%**	**4%**	**100%**

Table S11. Final Costs of the Clinical Departments of Mt Meru RH after Step-Down of Overhead Costs (in USD)

COST CENTRES	Final Costs				
	Recurrent		Capital		Total
	Personnel	Other recurrent	Medical equipment	Other capital	
**OPD/Casualty**	257,406	246,224		11,518	515,147
**Laboratory**	136,569	170,806	691	6,393	314,460
**Radiology**	75,047	38,292	6,047	2,682	122,069
**OG Theatre**	141,716	52,766	332	9,003	203,817
**Main Operating Theatre**	179,777	46,542	659	11,525	238,503
**Paediatric Ward**	267,202	31,552		25,834	324,589
**Maternity Wards + Gynae OPD**	310,679	104,233		52,722	467,634
**SOPD**	28,650	3,421		1,124	33,195
**Eye Department**	54,820	7,714		2,441	64,975
**Medical Ward + CTC + TB Unit**	357,289	53,427		36,923	447,639
**Surgical Ward**	321,067	90,511		28,972	440,550
**Dental Health**	57,939	10,082		1,742	69,763
**Physiotherapy + Mental Health + ICU**	178,375	20,371		12,315	211,060
**Total**	2,366,536	875,941	7,730	203,194	3,453,401
	**69%**	**25%**	**0.2%**	**6%**	**100%**

Table S12. Final Costs of the Clinical Departments at Meru DH after Step-Down of Overhead Costs (in USD)

COST CENTRES	Final Costs

	Recurrent		Capital		Total
	Personnel	Other recurrent	Medical equipment	Other capital	
**OPD**	200,712	416,706,946		9,433	396,415
**Laboratory**	82,811	9,890.09	8,750	2,335	103,786
**Radiology**	25,019	3,575.62	3,792	1,943	34,330
**Main Operating Theatre**	79,584	20,585.48	8,160	4,793	113,123
**Minor Operating Theatre**	67,922	17,413.61	641	3,157	89,133
**Paediatric Ward**	93,825	6,482.15		4,073	104,381
**Maternity Ward**	166,942	26,587.98		10,994	204,525
**Female Ward**	72,899	7,380.52		6,410	86,689
**Male Ward**	69,920	6,499.33		6,721	83,140
**RCH (Including CECAP)**	79,970	5,856.12		9,594	95,421
**Physiotherapy**	42,679	3,150.22		2,831	48,661
**CTC + TB/Leprosy Unit**	89,984	9,117.17		8,357	107,458
**Eye + Dental Units**	64,902	4,263.15		6,532	75,697
**Total:**	1,137,170	307,071.64	47,746,473	77,174	1,542,758
	**74%**	**20%**	**1%**	**5%**	**100%**

Table S13. Final Costs of the Clinical Departments at Oltrumet DH after Step-Down of Overhead Costs (in USD)

**COST CENTRES**	**Final Costs**				
	Recurrent		Capital		Total
	Personnel	Other recurrent	Medical equipment	Other capital	
**Out-Patient Department**	100,746	87,756		4,832	193,334
**Laboratory Department**	22,190	1,212	1,858	1,536	26,796
**Radiology**	11,093,932	431	388	523	6,301
**RCH**	27,205	1,887	1,250	2,248	32,590

**Main Operating Theatre**	36,024	8,742	3,671	5,655	54,092
**Minor Operating Theatre**	11,595	2,350		753	14,698
**Maternity Ward**	79,590	14,199		8,926	102,715
**Female Ward (+Paediatric) ward**	32,168	16,665		6,462	55,295
**Male Ward**	23,892	5,242		4,139	33,274
**Physiotherapy**	7,944	693		1,067	9,704
**CTC + TB Unit**	18,679	1,514		2,215	22,409
**Mental Health**	6,653	635		817	8,105
**Dental Unit**	12,446	1,055		2,744	16,245
**Total**	384,092	142,381	7,167	41,917	575,557
	**67%**	**25%**	**1%**	**7%**	**100%**

Table S14. Activity Profile per Type of Surgery at Mt Meru RH

INDEX SURGERIES	Pre-op consults	Pre-op stay (surgical)	Pre-op stay (Obs/Gynae)	Lab tests	Imaging tests	OT time (main OT)	OT time (Obs/Gynae)	Post-op stay (surgical)	Post-op stay (Obs/Gynae)	Follow -up
**Herniorrhaphy**	1	1	0	1	0	0.88	0	1	0	2
**Laparotomy**	0	0	0	1	1	2.25	0	4	0	2.5
**Appendectomy**	0	0.1	0	1	0	0.75	0	1	0	2
**Adenotonsillectomy**	2	1	0	1	0	0.63	0	0.2	0	1
**Tonsillectomy**	2	1	0	1	0	0.33	0	0.2	0	1
**Adenectomy**	2	1	0	1	0	0.33	0	0.2	0	1
**Surgical debridement**	0	0.33	0	1	1	2.00	0	24.5	0	4
**ORIF**	4	1	0	1	1	1.50	0	2	0	4
**CS**	0.5	0	0.5	1	1	0	0.75	0	1.5	2
**Hysterectomy**	4	0	2	1	1	0	1.25	0	2.5	2

**Myomectomy**	2	0	2	1	1	0	2.50	0	2.5	2
**Salpingectomy**	0	0	0	1	1	0	0.54	0	2.5	2

Table S15. Activity Profile per Type of Surgery at Meru DH

INDEX SURGERIES	Pre-op consults	Pre-op stay (surgical)	Pre-op stay (Obs/Gynae)	Lab tests	Imaging tests	OT time (main OT)	OT time (Obs/Gynae)	Post-op stay (surgical)	Post-op stay (Obs/Gynae)	Follow -up
**CS**	0.5	0	0.25	1	1	0.68	0	0	2.17	2.33
**Salpingectomy**	0	0	0	1	1	0.78	0	2.4	0	1.8
**Appendectomy**	0.1	0.1	0	1	1	0.75	0	1	0	2
**Herniorrhaphy**	1	1	0	1	0	1.19	0	2.75	0	2
**Myomectomy**	3.5	1	0	1	1	1.29	0	2.83	0	1.67
**Hydrocoelectomy**	1	1	0	1	0	0.33	0	2	0	2
**Other major surgeries**	0	0.33	0	1	1	2	0	2.5	0	4

Table S16. Activity Profile per Type of Surgery at Oltrumet DH

INDEX SURGERIES	Pre-op consults	Pre-op stay (surgical)	Pre-op stay (Obs/Gynae)	Lab tests	Imaging tests	OT time (main OT)	OT time (Obs/Gynae)	Post-op stay (surgical)	Post-op stay (Obs/Gynae)	Follow -up
**CS**	0.5	0	0.25	1	1	0.63	0	0	2.5	2
**Herniorrhaphy**	2	1	0	1	0	0.50	0	2	2	2
**Myomectomy**	4	1	0	1	1	1.88	0	1	2.5	2

**Other surgeries**	2	1	0	1	0	0.63	0	0.2	0	0

Abbreviations Used in Tables S1-S16: BIMA = National Health Insurance Fund; CECAP = Cervical Cancer Prevention; CS = Caesarean Section; CTC = Care and Treatment Centre; DH = District Hospital; DIVO = District Immunisation and Vaccination Office; DM = Diabetes Mellitus; ENT = Ear, Nose and Throat; GOPD = General Out-Patient Department; ICU = Intensive Care Unit; Intra-op = Intra-operative; Lab = Laboratory; M&E = Monitoring and Evaluation; MA = Medical Attendants; Mt = Mount; Obs/Gynae = Obstetrics and Gynaecology; OPD = Out-Patient Department; OT = Operating Theatre; POPD = Paediatric Out-Patient Department; Post-op = Post-operative; Pre-op = Pre-operative; RCH = Reproductive and Child Health; RH = Referral Hospital; SOPD = Surgical Out-Patient Department; TB = Tuberculosis; USD = United States Dollars.
